# Echocardiographic study of myocardial work in patients with type 2 diabetes mellitus

**DOI:** 10.1186/s12872-022-02482-3

**Published:** 2022-02-17

**Authors:** Lisi Liao, Bobo Shi, Zhimin Ding, Lixin Chen, Fajin Dong, Jian Li, Yulin Zhong, Jinfeng Xu

**Affiliations:** 1 Department of Ultrasound, First Affiliated Hospital of Southern University of Science and Technology, Shenzhen Medical Ultrasound Engineering Center, Shenzhen People’s Hospital, 1017 Dongmen North Road, Luohu District, Shenzhen, 518020 Guangdong China; 2 Department of Ultrasound, Second Clinical College of Jinan University, Shenzhen People’s Hospital, Shenzhen, China

**Keywords:** Diabetes, Echocardiography, Left ventricular pressure-strain loop, Myocardial work

## Abstract

**Background:**

A noninvasive left ventricular (LV) pressure-strain loop (PSL) provides a new method to quantify myocardial work (MW) by combining global longitudinal strain (GLS) and LV pressure, which exerts potential advantages over traditional GLS. We studied the LV PSL and MW in patients with type 2 diabetes mellitus (T2DM).

**Methods:**

This cross-sectional study included 201 subjects (54 healthy controls and 147 T2DM patients) who underwent complete two-dimensional echocardiography (2DE), including 2D speckle-tracking echocardiography (STE), as well as brachial artery pulse pressure measurement. The PSL was used to determine the global myocardial work index (GWI), global constructive work (GCW), global wasted work (GWW), and global work efficiency (GWE) of all study participants. The association between T2DM and LV function was evaluated according to these MW indices.

**Results:**

The GLS was significantly lower in the T2DM group than in the control group (*P* < 0.001), indicating that the LV myocardium had been damaged, although the LV ejection fraction (LVEF) was still normal. The GWI and GWE were decreased (*P* = 0.022) and the GWW was increased (*P* < 0.001) in diabetic patients compared with controls, but the GCW was comparable in the two groups (*P* = 0.160). In all diabetic patients, age, body mass index, systolic blood pressure, smoking history, and LVEF were correlated with GWI, GWW and GWE.

**Conclusions:**

The use of LV PSL is a novel noninvasive technique that could help to depict the relationship between LV myocardial damage and MW in patients with T2DM.

## Background

Type 2 diabetes mellitus (T2DM) is a global epidemic. The global prevalence of diabetes in 2019 was estimated to be 9.3% (463 million) and is expected to increase to 10.2% (578 million) by 2030 and 10.9% (700 million) by 2045. T2DM accounts for approximately 90% of the total, with an estimated number of cases of approximately 520 million by 2030 and 630 million by 2045 [1, 2].

Studies have shown that diabetes can lead to the development of structural heart disease and heart failure (HF) through systemic, myocardial and cellular mechanisms [3]. A review of recent studies has detailed the underlying mechanisms of diabetes-related HF [4], emphasizing that hyperglycemia and hyperinsulinemia accelerate atherosclerosis through vascular smooth muscle cell (VSMC) proliferation and inflammation. In a recent study, Daniele Torella et al. showed that miR-29c overexpression and miR-204 inhibition fostered a reduction in the phenotypic switch of VSMCs in T2DM [5]. Diabetes is associated with atherogenic dyslipidemia, in which low-density lipoprotein (LDL) cholesterol particles are likely to cause atherosclerosis and are associated with endothelial dysfunction, which promotes leukocyte and platelet adhesion, thrombosis, inflammation, and coronary plaque ulceration [6]. Therefore, T2DM is a risk factor for cardiovascular diseases, such as myocardial ischemia and infarction [7]. At the same time, the risk of HF in diabetic patients was more than twice that in nondiabetic patients. Moreover, compared with patients without diabetes, patients with diabetes have worse cardiovascular outcomes, higher rates of hospitalization and a poorer prognosis [8]. In fact, tight glycemic control during acute coronary syndromes reduces the mortality rate and improves the prognosis of patients with and without diabetes [9, 10]. Cardiovascular diseases worsen the quality of life of patients with T2DM and increase their medical costs. As a result, the early detection of cardiovascular disease in patients with T2DM is essential to improve outcomes in this population.

There are three kinds of cardiac abnormalities in diabetic heart disease: left ventricular (LV) systolic dysfunction, diastolic dysfunction and LV geometric changes. Echocardiography is the most commonly used method to examine the cardiac function of patients with DM. Determination of the ejection fraction (EF) and speckle tracking are commonly used to evaluate cardiac function. Although a decreased EF is considered a sign of HF in symptomatic patients, most patients with HF are still in the stage of EF preservation [11, 12], which cannot be efficiently detected by traditional echocardiographic parameters. The LV global longitudinal strain (GLS) is a load-dependent measurement of LV function; however, its accuracy in evaluating LV function under certain hemodynamic conditions is challenging [13]. Therefore, developing noninvasive technologies to evaluate myocardial lesions in diabetic patients is particularly important.

Myocardial work (MW) is a set of parameters related to afterload that overcomes the load dependence of the LVEF and LV strain. It is a dynamic noninvasive method that considers myocardial deformation and blood pressure (BP). This method was developed nearly 30 years ago, but it takes into account the invasive measurements obtained during cardiac catheterization, which is impractical in clinical practice. The global myocardial work index (GWI), global constructive work (GCW), global wasted work (GWW), and global work efficiency (GWE) are MW parameters that are derived from the analysis of the LV pressure-strain loop (PSL) combined with noninvasive BP and strain [14–17]. Although MW has shown great clinical potential, there have been few studies on LV MW in diabetic patients.

Therefore, the aim of this study was (1) to describe global indices of MW in healthy individuals and patients with T2DM; (2) to assess the correlation between MW and other basic and echocardiographic parameters; and (3) to evaluate the association of MW and adverse outcomes to determine the degree of myocardial damage in patients with T2DM and to monitor and protect myocardial function.

## Methods

### Study population

This was a cross-sectional study. Patients who underwent echocardiography in the Department of Ultrasound, Shenzhen People's Hospital from August 2020 to May 2021 were included.

The inclusion criteria were as follows: recent diagnosis (≤ 3 months) of T2DM or poor glycemic control (glycosylated hemoglobin (HbA1c) ≥ 6.5% and fasting plasma glucose ≥ 7.0 mmol/L).

The exclusion criteria were as follows: HF; coronary artery disease (coronary artery disease was identified by medical history, coronary computed tomography angiography, echocardiography, invasive coronary imaging, electrocardiography, cardiopulmonary exercise test, and clinical symptoms of coronary artery disease); moderate or above valvular regurgitation disease; atrial fibrillation; tumor; liver cirrhosis or renal failure; hypertension with poor BP control; and poor echocardiogram image quality.

Basic data were obtained from all participants and included sex, age, height, weight, body mass index (BMI), smoking history, BP, T2DM duration and laboratory examination data [fasting blood glucose (mmol/L), triglyceride (mmol/L), high-density lipoprotein (HDL, mmol/L), LDL (mmol/L), total cholesterol (mmol/L), creatinine (µmol/L), and HbA1c (%)]. T2DM was diagnosed according to current clinical guidelines. Data related to drug use were obtained from all participants in the study. The study was approved by the local ethics committee of our hospital, and written informed consent was obtained from all participants.

### BP measurement

BP measurement for each patient was performed in a quiet 25 °C room after a 10-min supine rest. We measured the brachial artery BP and heart rate in the right arm with an automatic digital sphygmomanometer (OMRON, Kyoto, Japan). BP was measured before and immediately after echocardiography, and the mean value was used for statistical analysis.

### Echocardiography

Echocardiography was performed with a model E95 ultrasound machine (GE Healthcare). All parameters were the average of three consecutive cardiac cycles. The left atrial dimension (LAD), LV end-diastolic dimension (LVEDD), LV end-systolic dimension (LVESD), LV end-diastolic volume (LVEDV), LV end-systolic volume (LVESV), interventricular septum (IVS) thickness, posterior wall thickness (PWT), and LV stroke volume (LVSV) were obtained according to the guidelines of the American Society of Echocardiography [18]. The LVEF was evaluated by the biplane method, and Doppler blood flow and tissue Doppler velocity imaging were used to obtain the E wave (cm/s), A wave (cm/s), E/A ratio, septal e′ wave (m/s), lateral e′ wave (m/s), and E/e′ ratio.

### LV strain analysis and MW analysis

Three consecutive cardiac cycles were collected for two-dimensional (2D) strain analysis [18]. Q-analysis (echoPAC version 202, GE Healthcare) was used to evaluate 2D strain. Data were collected from three apical views (3, 4 and 2 chambers). The GLS of the LV myocardium was evaluated on the apical view. The performing physician verified the endocardial contour automatically tracked by the software and adjusted the region of interest to confirm that all images included the entire LV wall thickness. The left ventricle was divided into 17 segments to calculate the longitudinal strain.

**Fig. 1:**
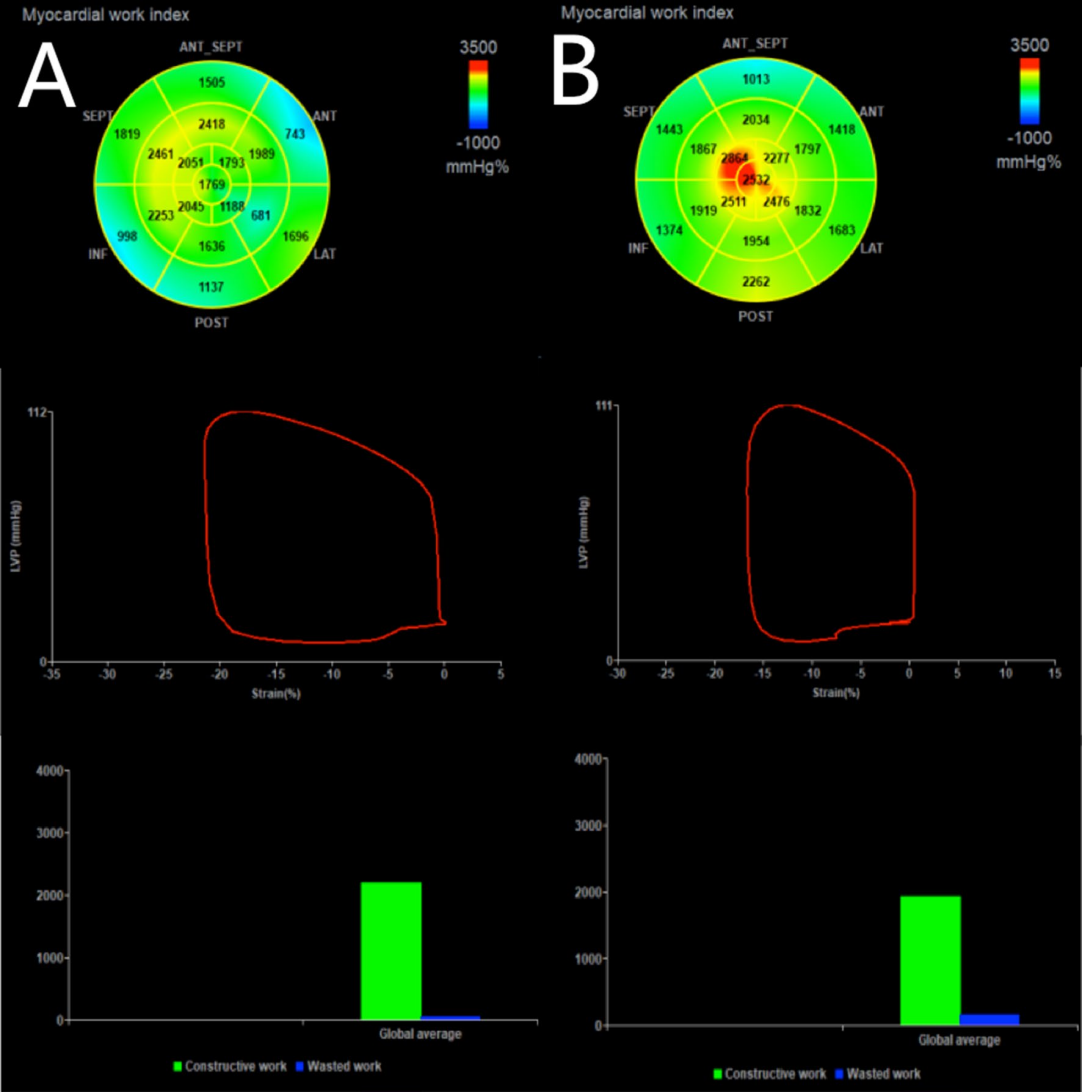
(Top panel) Seventeen-segment bull’s-eye representation of MW index (GWI) showing areas of negative work in blue, normal in green, and red indicating areas of high MW; (Middle panel) LV PSL; (Bottom panel) Green bar shows constructive work; Blue bar shows wasted work. Examples of patients within: **A** control group; **B** T2DM

MW was evaluated using the same software. After calculating the GLS of the left ventricle, the brachial artery BP was entered, and the valve opening and closing time was determined by echocardiography [19, 20]. The timing of valve opening and closing was determined by spectral Doppler imaging at the mitral and aortic valve levels. The software provides a noninvasive PSL by integrating GLS, BP and valve opening and closing timing data. The annular area, as does the GWI, corresponds to the total work from mitral valve closure to mitral valve opening in the LV pressure-strain annular area. In addition, other indices of MW were calculated (Fig. 1), as follows: GCW (work done by systolic LV shortening and diastolic LV lengthening); GWW (work done by systolic LV lengthening and diastolic LV shortening); and GWE (effective work divided by the sum of effective work and ineffective work). All studies were analyzed by two ultrasound physicians (LLS and SBB) blinded to the clinical and laboratory data (double-blinded analysis).

### Statistical analysis

Continuous variables are represented as the means ± standard deviations and were compared using independent sample t-tests. Age, BMI and systolic BP (SBP) were used as covariates, and multivariate correlation analysis was used to evaluate the relationship of basic, laboratory and echocardiographic parameters with MW parameters. Interobserver and intragroup correlation coefficients (ICCs) were used to assess repeatability. *P* < 0.05 was considered statistically significant.

## Results

### Study population

This study population consisted of 201 individuals: 147 patients with T2DM (mean age: 51.35 ± 12.06 years; 66% male) and 54 healthy controls (mean age: 48. 33 ± 9.88 years; 53% male). The clinical characteristics of the patients in the two groups are shown in Table 1. There was no significant difference in age or sex between the two groups, but there was a significant difference in BMI, smoking history, SBP and diastolic BP (DBP) (*P* < 0.05). In terms of laboratory parameters, there were significant differences in fasting blood glucose, HDL, LDL, triglycerides, and HbA1c between the groups; the differences in fasting blood glucose, HDL, triglycerides and HbA1c were all significant with *P* < 0.01.

**Table 1:** Baseline characteristics of two groups

	Controls (n = 54)	T2DM (n = 147)	*P* value
Age (years)	48.33 ± 9.88	51.35 ± 12.06	0.101
Male (%)	53	66	0.075
BMI (kg/m^2^)	22.93 ± 3.33	27.04 ± 5.59	< 0.001**
SBP (mmHg)	130.01 ± 11.20	132.03 ± 18.67	< 0.001**
DBP (mmHg)	76.05 ± 8.10	83.15 ± 10.80	< 0.001**
Smoker (%)	1.8	38	< 0.001**
Fasting glucose level (mmol/L)	5.02 ± 0.61	7.75 ± 2.61	< 0.001**
HbA1c (%)	5.46 ± 0.58	9.96 ± 8.09	< 0.001**
Triglycerides (mmol/L)	1.40 ± 0.80	2.31 ± 2.31	0.005**
TC (mmol/)	5.02 ± 0.61	4.80 ± 1.33	0.245
Scr (umol/L)	71.93 ± 16.86	80.30 ± 38.15	0.122
HDL (mmol/L)	1.31 ± 0.36	1.07 ± 0.30	< 0.001**
LDL (mmol/L)	3.10 ± 0.64	2.75 ± 0.98	0.016*

T2DM, type 2 diabetes mellitus; BMI, body mass index; SBP, systolic blood pressure; DBP, diastolic blood pressure; HbA1c, glycosylated hemoglobin; TC, Triglycerides; Scr, Serum creatinine; HDL, High density lipoprotein; LDL, Low density lipoprotein

*P < 0.01

**P < 0.05

### Results of traditional echocardiography

Table 2 presents the echocardiographic characteristics of the diabetic patients and healthy controls. The LAD was greater in the diabetic patients than in the controls (32. 93 ± 3.85 vs. 30.14 ± 3.89 mm; *P* < 0.001), and the IVS thickness and PWT were greater (10.48 ± 1.46 vs. 8.53 ± 1.25 mm, 9.94 ± 1.59 vs. 9.94 ± 1.59; *P* < 0.001). There was a significant difference in the E/A ratio and E/e' ratio between the control and T2DM groups (1.42 ± 0.40 vs. 0.88 ± 0.32; 7.09 ± 1.69 vs. 9.64 ± 3.24). Although the LV volume of diabetic patients was slightly lower and LV diastolic function was more impaired, there was no significant difference in the LVEDD, LVESD, LVEDV, LVESV, or LVEF between the diabetic patients and controls.

**Table 2:** Echocardiographic parameters of left ventricular structure and function in the study population

	Controls (n = 54)	T2DM (n = 147)	*P* value
LAD (mm)	30.14 ± 3.89	32.93 ± 3.85	< 0.001**
LVEDD (mm)	46.29 ± 4.90	45.46 ± 5.11	0.306
LVESD (mm)	29.11 ± 4.14	28.88 ± 4.11	0.730
IVS (mm)	8.52 ± 1.25	10.28 ± 1.46	< 0.001**
PWT (mm)	8.29 ± 1.32	9.94 ± 1.59	< 0.001**
LVEDV (mL)	100.59 ± 24.89	96.62 ± 25.72	0.330
LVESV (mL)	32.14 ± 10.66	21.93 ± 12.19	0.677
LVSV (mL/m^2^)	67.88 ± 16.96	63.58 ± 17.70	0.125
LVEF (%)	67.69 ± 5.28	66.18 ± 6.13	0.062
E/A ratio	1.42 ± 0.40	0.88 ± 0.32	< 0.001**
E/e′	7.09 ± 1.69	9.64 ± 3.24	< 0.001**
LVDF, normal	54/54	49/147	< 0.001**

T2DM, type2 diabetes mellitus; LAD, left atrial dimension; LVEDD, left ventricular end-diastolic dimension; LVESD, left ventricular end-systolic dimension; IVS, interventricular septum; PWT, posterior wall thinkness, LVEDV, left ventricular end-diastolic volume; LVESV, left ventricular end-systolic volume; LVSV, left ventricular stroke volume; LVEF, left ventricular ejection fraction; A, late diastolic mitral flow (pulse Doppler); E, early diastolic mitral flow (pulse Doppler); mitral annulus(tissue Doppler); e′, average of the peak early diastolic relaxation velocity of the septal and lateral; LVDF, left ventricular diastolic function

**P < 0.01

*P < 0.05

### LV GLS and LV MW

Table 3 summarizes the LV GLS and MW data. Compared with the controls, the LV GLS was significantly impaired in the diabetic patients (− 16.82 0 ± 2.59% vs. − 19.13 ± 1.72%; *P* < 0.001). The GWI in the control and T2DM groups was 1692.86 ± 349.63 vs. 1814.50 ± 270.50 mmHg% (*P* < 0.05). There was no significant difference in the GCW between the control and T2DM groups (1998. 76 0 ± 362.53 vs. 2075.13 ± 296.14 mmHg%, *P* = 0.160), and the GWW was significantly greater in the T2DM group than in the control group (140 ± 87.63 mmHg% [IQR: 126.67–155.24 mmHg%] vs. 69.22 ± 31.49 mmHg% [IQR: 60.63–77.82 mmHg%]; *P* < 0.001). This condition led to a decrease in GWE; the GWE in the T2DM group was 92.26% ± 28% (IQR: 91.56–92.69%), while that in the control group was 96.07% ± 1.52% (IQR: 95.66–96.49%) (*P* < 0.001).

**Table 3:** Speckle-tracking and myocardial work assessment of left ventricular function in the study population

	Controls (n = 54)	T2DM (n = 147)	*P* value
GLS, %	− 19.13 ± 1.73	− 16.82 ± 2.59	< 0.001**
Myocardial work			
GWI (mmHg%)	1814.50 ± 270.50	1692.86 ± 349.63	0.022*
GCW (mmHg%)	2075.13 ± 269.14	1998.76 ± 362.53	0.160
GWW (mmHg%)	69.22 ± 31.49	140.96 ± 87.63	< 0.001**
GWE (%)	96.07 ± 1.52	92.26 ± 4.28	< 0.001**

T2DM, type2 diabetes mellitus; GLS, left ventricular global longitudinal strain; GWI, global myocardial work index; GCW, global constructive work; GWW, global wasted work; GWE, global myocardial work efficiency

**P < 0.01

*P < 0.05

### Correlation between MW and other parameters

GWI was correlated with triglycerides (β = − 0.13, *P* < 0.05), sex (β = − 0.19, *P* < 0.01), age (β = 0.19, *P* < 0.01), BMI (β = − 0.18, *P* = 0.01), smoking history (β = − 0.28, *P* < 0.01), SBP (β = 0.34, *P* < 0.01), fasting blood glucose (β = − 0.271, *P* < 0.01), HDL (β = 0.20, *P* < 0.01), and LVEF (β = − 0.23, *P* = 0.001, Table 4).

**Table 4:** multivariate analyses of echocardiographic parameters and myocardial function parameters in patients with diabetes mellitus

	GWI (mmHg%)		GWW (mmHg%)		GWE (%)	
	β	*P*	β	*P*	β	*P*
Age (years)	0.197	0.005**	0.282	< 0.001**	− 0.221	0.002**
Male (%)	− 0.188	0.007**	0.004	0.954	− 0.069	0.327
BMI (kg/m^2^)	− 0.181	0.010**	0.156	0.027*	− 0.238	0.001**
Smoker	− 0.281	< 0.001**	0.170	0.016*	0.485	< 0.001**
SBP (mmHg)	0.343	< 0.001**	0.409	< 0.001**	− 0.333	< 0.001**
T2DM duration (years)	0.198	0.016*	0.062	0.459	0.018	0.832
DBP (mmHg	0.025	0.724	0.206	0.003**	− 0.231	0.001**
Fasting glucose level (mmol/L)	− 0.271	< 0.001**	0.132	0.063	− 0.204	0.004**
HbA1c (%)	− 0.041	0.572	0.097	0.174	− 0.099	0.165
Triglycerides (mmol/L)	− 0.139	0.049*	0.043	0.549	− 0.118	0.098
TC (mmol/L)	0.018	0.799	− 0.014	0.841	0.007	0.926
Scr (umol/L)	− 0.026	0.717	0.081	0.259	− 0.179	0.012*
HDL (mmol/L)	0.203	0.004**	− 0.103	0.146	0.189	0.007**
LDL (mmol/L)	0.090	0.207	− 0.093	0.190	0.107	0.134
LAD (mm)	0.006	0.933	0.234	0.001**	− 0.282	< 0.001**
LVEDD (mm)	0.009	0.901	0.105	0.139	− 0.135	0.057
LVESD (mm)	− 0.094	0.187	0.132	0.061	− 0.210	0.003**
IVS (mm)	− 0.065	0.362	0.404	< 0.001**	− 0.415	< 0.001**
PWT (mm)	− 0.127	0.072	0.361	< 0.001**	− 0.401	< 0.001**
LVEDV (mL)	0.002	0.979	0.117	0.099	− 0.148	0.037*
LVESV (mL)	− 0.132	0.062	0.166	0.018*	− 0.260	< 0.001**
LVSV (mL/m^2^)	0.069	0.332	0.052	0.464	− 0.032	0.651
LVEF (%)	0.237	0.001**	− 0.133	0.060*	0.233	0.001**
E/A ratio	0.053	0.452	− 0.339	< 0.001**	0.350	< 0.001**
E/e′ ratio	0.126	0.075	0.275	< 0.001**	− 0.270	< 0.001**
LVDF	− 0.059	0.409	0.533	< 0.001**	− 0.552	< 0.001**

T2DM, type2 diabetes mellitus; BMI, body mass index; SBP, systolic blood pressure; DBP, diastolic blood pressure; HbA1c, glycosylated hemoglobin; TC, Triglycerides; Scr, Serum creatinine; HDL, High density lipoprotein; LDL, Low density lipoprotein; LAD, left atrial dimension; LVEDD, left ventricular end-diastolic dimension; LVESD, left ventricular end-systolic dimension; IVS, interventricular septum; PWT, posterior wall thickness; LVEDV, left ventricular end-diastolic volume; LVESV, left ventricular end-systolic volume; LVSV, left ventricular-stroke volume; LVEF, left ventricular ejection fraction; A, late diastolic mitral flow(pulse Doppler); E, early diastolic mitral flow(pulse Doppler); e′, mitral annulus(tissue Doppler); average of the peak early diastolic relaxation velocity of the septal and lateral; LVDF, left ventricular diastolic function

**P < 0.01

*P < 0.05

GWW was correlated with BMI (β = − 0.15, *P* < 0.05), smoking history (β = − 0.17, *P* < 0.05), LVESV (β = 0.166, *P* < 0.05), age (β = 0.28, *P* < 0.01), SBP (β = 0.40, *P* < 0.01), DBP (β = 0.20, *P* < 0.01), LAD (β = − 0.23, *P* = 0.001), IVS thickness (β = 0.40, *P* < 0.01), LV PWT (β = 0.36, *P* < 0.01), E/A ratio (β = − 0.33, *P* < 0.01), E/e' ratio (β = − 0.27, *P* < 0.05), LV PWT (β = 0.36, *P* < 0.01), and LVEF (β = 0.53, *P* < 0.01).

GWE was correlated with LVEDV (β = − 0.14, *P* < 0.05), creatinine (β = − 0.17, *P* < 0.05), BMI (β = − 0.23, *P* = 0.001), age (β = − 0.22, *P* < 0.01), smoking history (β = − 0.485, *P* < 0.01), SBP (β = − 0.33, *P* < 0.01), DBP (β = − 0.23, *P* = 0.001), fasting blood glucose (β = − 0.20, *P* < 0.01), HDL (β = 0.18, *P* < 0.01), LAD (β = − 0.28, *P* < 0.01), LVESD (β = − 0.21, *P* < 0.01), IVS thickness (β = − 0.41, *P* < 0.01), PWT (β = − 0.40, *P* < 0.01), LVESV (β = − 0.26, *P* < 0.01), LVEF (β = − 0.23, *P* = 0.001), E/A ratio (β = 0.35, *P* < 0.01), E/e' ratio (β = − 0.27, *P* < 0.01), and LVEF (β = − 0.55, *P* < 0.01). Age, BMI, and SBP were used as covariates after correction, and there were still significant differences.

T2DM duration was associated with E/A ratio (β = − 0.31, *P* < 0.01), E/e' ratio (β = 0.45, *P* < 0.01), LV diastolic function (β = 0.36, *P* < 0.01), GWI (β = 0.19, *P* < 0.05) and GCW (β = 0.25, *P* < 0.01). Moreover, there were no correlations with other echocardiographic parameters.

### Intraobserver and interobserver variability of MW parameters

The interclass correlation coefficients (ICCs) for interobserver variability were as follows: GLS, 0.97 (95% CI: 0.96–0.99; *P* < 0.01); GWI, 0.95 (95% CI: 0.90–0.97; *P* < 0.01); GCW, 0.93 (95% CI: 0.89–0.96; *P* < 0.01); GWW, 0.82 (95% CI: 0.68–0.94; *P* < 0.01); and GWE, 0.88 (95% CI: 0.55–0.96; *P* < 0.01).

The ICCs for intraobserver variability were as follows: GLS, 0.98 (95% CI: 0.92–0.99; *P* < 0.01); GWI, 0.94 (95% CI: 0.85–0.96; *P* < 0. 01); GCW, 0.91 (95% CI: 0.83–0.95; *P* < 0.01); GWW, 0.82 (95% CI: 0.55–0.90; *P* < 0. 05); and GWE, 0.88 (95% CI: 0.67–0.94; *P* < 0.01).

## Discussion

The main findings of this study are as follows: (1) The GLS was lower in the T2DM group than in the control group, indicating that the myocardial contractile function of diabetic patients had changed while the LVEF was preserved. (2) Compared with the control group, the GWI was significantly reduced in the T2DM group, but the GCW showed no significant difference, and the GWW was significantly greater in the T2DM group, which led to a decrease in the GWE in diabetic patients. (3) Age, BMI, SBP, smoking history, and LVEF were associated with GWI, GWW, and GWE in patients with diabetes.

Cardiovascular disease is the main cause of death in T2DM [21]. Diabetes can cause major vascular complications (cardiovascular disease) and microvascular complications (e.g., diabetic nephropathy, diabetic retinopathy, and neuropathy), which increase mortality, blindness, and renal failure in diabetic patients [22]. Therefore, some echocardiographic methods have been proposed to better evaluate the LV function of diabetic patients. In the past few years, determination of the LV GLS from strain tracking has become a daily measurement modality to evaluate LV function in diabetic patients and has shown a good correlation with histology-confirmed myocardial fibrosis [23, 24]. The GLS was lower in the T2DM group than in the control group, indicating that patients with diabetes have impaired myocardial contractility even when the LVEF is in the normal range. A decreased GLS is also common in asymptomatic diabetic patients, although the incidence is lower than that of diastolic dysfunction [11]. An abnormal GLS is related to the development of HF, mortality, and LV remodeling [12]. The reduced strain rate is a sign of contractility reduction, and the underlying causes include insulin signal changes, apoptosis, and necrosis leading to the loss of contractile cells and stress on the endoplasmic reticulum [25].

LV GLS is still dependent on load, which may be a limitation in the event of changes in hemodynamic conditions. Myocardial function has been introduced as a new LV function parameter that takes into account LV deformation and LV afterload. Based on noninvasive LV pressure (BP) measurement, the LV PSL was constructed. Compared with the traditional GLS method, this approach combines measurement of the myocardial deformation and stress load and has advantages [14, 26, 27]. Russell et al. also validated this method by invasive LV pressure measurements, and the LV PSL area was strongly correlated with myocardial metabolism during positron emission tomographic assessment [28].

The GWI, GCW, GWW, and GWE in the control group were consistent with the normal values obtained in the European Association of Cardiovascular Imaging (EACVI) Normal Reference Ranges for Echocardiography (NORRE) study. Our study also confirms the results of other previous studies [29]. In this study, the GWI was significantly lower in the T2DM group than in the control group, and while there was no difference in the GCW, the GWW was significantly increased, which led to a decrease in the GWE in the T2DM group. It is suggested that even if the LVEF is preserved, the GWI has already decreased, the GWW has significantly increased, and the GWE has decreased. LV dysfunction can occur in the early stage of myocardial impairment in diabetic patients. The hypothesis of diabetic myocardial disease includes changes in myocardial calcium homeostasis, mitochondrial dysfunction, oxidative stress, direct glucotoxicity, myocardial fibrosis, disorder of myocardial matrix use, and myocardial lipid accumulation [30, 31]. Specifically, T2DM is related to myocardial fibrosis or collagen content and myocardial stiffness [32, 33]. Fibrosis of the endocardium, which causes focal fibrosis and low perfusion caused by the redistribution of myocardial blood flow due to its contraction, is impaired by myocardial deformation [34], which leads to a decrease in the GWI, an increase in the GWW, and subsequently a decrease in the GWE.

In this study, there was a significant difference in smoking among the baseline data between the control and T2DM groups. After multivariate regression analysis, smoking was significantly correlated with the GWI, GWW and GWE, indicating that smoking impairs myocardial function. There is evidence that nicotine can cause direct damage to the structure and function of the heart even in patients without atherosclerosis or other chronic diseases related to the risk of cardiovascular disease [35], and the amount of smoking is positively correlated with the degree of impairment of cardiac systolic function [36]. In the Multi-Ethnic Study of Atherosclerosis (MESA) study, it was found that the LV mass and geometry were associated with the occurrence of HF, stroke and coronary heart disease. Smoking can increase the LV mass/volume ratio and lead to a poor prognosis in patients with coronary heart disease [37].

In our study, SBP was significantly correlated with GWI, GWW and GWE, and DBP was significantly correlated with GWW and GWE, indicating that hypertension is associated with impaired myocardial function. Essential hypertension and T2DM are two common chronic diseases. T2DM can aggravate LV diastolic dysfunction in patients with essential hypertension [38, 39].

A recent study showed that subclinical myocardial dysfunction can be detected by real-time three-dimensional echocardiography (RT-3DE) in T2DM patients with poor glycemic control and that myocardial dysfunction is related to the duration of diabetes and level of HbA1c [40]. However, in our study, HbA1c showed no significant association with any echocardiographic parameter or MW index. It is speculated that this might be due to the small sample size of our study.

Gulsin GS et al. found that in asymptomatic working-age adults with T2DM, after weight loss, blood glucose control improved LV diastolic function, concentric LV remodeling and aortic stiffness, which provides the possibility that LV function in T2DM patients may be improved once metabolic control is obtained [41].

Recent data on diabetic patients showed that the use of either a glucagon-like peptide-1 receptor agonist (liraglutide) alone or in combination with a sodium-glucose cotransporter-2 inhibitor (empagliflozin) increased a number of cardiac work indicators in patients after 4 months of treatment and reduced cardiac failure compared to baseline (*P* = 0.041 and *P* < 0.001, respectively). All patients had an increase of MW index after 12 months of treatment [42]. It is suggested that LV myocardial function can be helpful in evaluating the myocardial condition of diabetic patients before and after treatment.

The introduction of cardiac parameters in the routine assessment of T2DM may improve the detection rate of early-stage heart disease in these patients. This **i**s of particular importance in diabetic patients, as cardiovascular disease may change with the course of diabetes, use of medication, and level of blood glucose control. This approach could serve as a better tool for clinicians to improve the follow-up of LV function in these patients and assess the potential effects of different treatments. In addition, it may represent a new risk stratification tool for assessing the prognosis of patients with T2DM.

### Limitations

There are several limitations to this study. First, the sample size was relatively small. In addition, the average age of the subjects was approximately 50 years. Thus, the generalization of our findings to other populations may require further confirmation. Second, we excluded some elderly patients upon speckle-tracking analysis failure, which may contribute to the risk of reporting bias. Third, we selected only patients with poorly controlled plasma glucose levels, leading to an increased risk of selection bias in this study. Finally, the study followed a cross-sectional design. Future studies with larger sample sizes following a cohort or randomized controlled trial design are needed to confirm our findings.

## Conclusions

This study shows that the LV PSL method could be used to evaluate myocardial dysfunction in T2DM patients in the early stage. The GWW of the patients was significantly increased, and the GWI and GWE were decreased, indicating that myocardial contractile function was damaged even while the LVEF was preserved. Age, BMI, SBP, smoking history and LVEF were all independently related to MW. Future studies with larger sample sizes evaluating the prognostic impact of MW on cardiovascular outcomes in diabetes are needed.

## Data Availability

The datasets generated and/or analyzed during the current study are not publicly available, but are available from the corresponding author on reasonable request.

## Abbreviations

T2DM: Type 2 diabetes mellitus
LV: Left ventricular
PSL: Pressure Strain Loop
MW: Myocardial work
GLS: Global longitudinal strain
2DE: Two-dimensional echocardiography
STE: Speckle tracking echocardiography
HF: Heart failure
BMI: Body mass index
SBP: Systolic blood pressure
DBP: Diastolic blood pressure
HbA1c: Glycosylated hemoglobin
HT: Hypertension
LAD: Left atrial dimension
LVEDD: Left ventricular end-diastolic dimension
LVESD: Left ventricular end-systolic dimension
IVS: Interventricular septum
PWT: Posterior wall thinkness
LVEDV: Left ventricular end-diastolic volume
LVESV: Left ventricular end-systolic volume
LVSV: Left ventricular-stroke volume
LVEF: Left ventricular ejection fraction
A: Late diastolic mitral flow (pulse Doppler)
E: Early diastolic mitral flow (pulse Doppler)mitral annulus(tissue Doppler)
e′: Average of the peak early diastolic relaxation velocity of the septal and lateral
LVDF: Left ventricular diastolic function
GLS: Left ventricular global longitudinal strain
GWI: Global myocardial work index
GCW: Global constructive work
GWW: Global wasted work
GWE: Global myocardial work efficiency
